# Modeling and Experimental Validation of Gradient Cell Density in PMMA Microcellular Foaming Induced by One-Sided Heating

**DOI:** 10.3390/polym17131780

**Published:** 2025-06-27

**Authors:** Donghwan Lim, Kwanhoon Kim, Jin Hong, Sung Woon Cha

**Affiliations:** Department of Mechanical Engineering, Yonsei University, 50 Yonsei-ro, Seodaemun-gu, Seoul 03722, Republic of Korea; imdh@yonsei.ac.kr (D.L.); kimkevin99@yonsei.ac.kr (K.K.); jin.hong@yonsei.ac.kr (J.H.)

**Keywords:** microcellular foaming, gradient cell density, nucleation theory, one-sided heating

## Abstract

Traditionally, the microcellular foaming process has aimed to generate uniform cell structures by applying heat uniformly to all surfaces of a polymer. Homogeneous cell distribution is known to enhance the mechanical properties and durability of the final product. However, the ability to engineer a gradient in cell density offers potential advantages for specific functional applications, such as improved sound absorption and thermal insulation. In this study, a controlled thermal gradient was introduced by heating only one side of a fully CO_2_-saturated poly(methyl methacrylate) (PMMA) specimen. This approach allowed for the formation of a cell density gradient across the sample thickness. The entire process was conducted using a solid-state batch foaming technique, commonly referred to as the microcellular foaming process. A one-sided heating strategy successfully induced a spatial variation in cell morphology. Furthermore, a coalescence function was developed to account for cell merging behavior, enabling the construction of a predictive model for local cell density. The proposed model accurately captured the evolution of cell density gradients under asymmetric thermal conditions and was validated through experimental observations, demonstrating its potential for precise control over foam structure in saturated PMMA systems.

## 1. Introduction

Extensive research has been conducted in various fields, including the automobile and information technology industries, to achieve lightweight structures via polymer foaming while minimizing the reduction in mechanical properties. In particular, chemical and physical foaming methods have been extensively investigated. However, chemical foaming is increasingly being regulated and phased out because of its negative impact on both the environment and human health, which is primarily caused by the emission of volatile organic compounds [1]. In addition, the resulting cell size typically exceeds 100 µm and is nonuniformly distributed, resulting in inconsistent mechanical properties, which create challenges for industrial applications. To address these limitations of chemical foaming, physical foaming using carbon dioxide (CO_2_) and nitrogen (N_2_) was developed in the late 1980s at the Massachusetts Institute of Technology (MIT) by N. P. Suh using solid-state batch foaming, which led to the development of the microcellular foaming process (MCP) [2,3]. When polymer foaming is performed using the MCP, microcellular foams with cell sizes smaller than 100 µm and cell densities exceeding 10^9^ cells/cm^3^ are formed within the polymer matrix [4]. This technology enables the lightweighting of polymers while maintaining minimal reductions in mechanical properties such as tensile and flexural strength, owing to the presence of numerous microcells. In addition, the impact strength can be several times higher than that of solid polymers [5,6,7,8]. Furthermore, when supercritical microcellular foaming is performed using injection molding with supercritical CO_2_ and molten polymers, post-molding shrinkage is significantly minimized, resulting in excellent dimensional stability. The reduced mold closing pressure reduces the energy consumption per unit during production, and the decrease in residual stress reduces the dimensional changes over time. Collectively, these benefits enable the efficient mass production of high-quality parts [9,10]. Given these advantages, microcellular foamed polymers are used in automotive components, such as door panels, dashboards, glove boxes, and brackets, which require both high strength and impact resistance. Their excellent impact strength also makes them well-suited for repeated use, leading to their use in home appliances such as refrigerators, washing machines, and printers. In particular, when used in helmets, microcellular foamed polymers offer significant weight reduction, which helps alleviate strain on the driver’s neck while effectively protecting the brain upon impact. These advantages make them particularly suitable for helmet applications [11]. However, a notable drawback is the difficulty in producing a smooth and visually uniform surface without external coating. As the gas-saturated polymer is injected into the mold and comes into contact with the mold surface, silver streaks may appear because of the presence of gas, which affects the overall surface quality of the final product [12]. This is a major drawback of the continuous MCP, and both raw material manufacturers and injection molding equipment companies are actively researching various methods to address and improve this issue [9,10].

Research based on injection molding requires various equipment, such as molds, injection machines, and gas injectors, and is not ideal for investigating cell behavior due to the aforementioned limitations. Therefore, in this study, a solid-state batch foaming process was employed using only a batch chamber and a high-pressure gas cylinder. In the solid-state batch foaming method, inert gas is introduced into a solid polymer using a high-pressure chamber, allowing the gas to saturate the free volume between the polymer chains. Subsequent thermodynamic instability, which is induced by heating or depressurization, triggers cell nucleation and growth, resulting in the formation of numerous cells within the polymer. CO_2_ and N_2_ are commonly used as inert gases because of their ability to avoid leaving residual solvents after the MCP and their environmentally friendly properties.

In previous studies, gradient cell densities were typically realized by designing asymmetric gas concentration profiles, followed by uniform heating or depressurization across the entire specimen [13,14]. Furthermore, research has also been conducted to generate a gradual variation in pore size distribution by applying different cooling rates to two surfaces [15]. Alternatively, a two-step foaming strategy can be used to induce gradient foams [16]. However, in this study, a gradient foam structure was achieved by applying one-sided heating to a fully saturated poly(methyl methacrylate) (PMMA) sheet. This approach offers a significant advantage in that it enables the formation of a gradient cell density through a simple method—fully saturating the polymer with gas followed by localized one-sided heating—without the need for precise control of gas concentration gradients or depressurization rates, which are often difficult to achieve in other studies. By creating a gradient foam, the spatial distribution of cells can be tailored to enhance the impact strength in the targeted regions and optimize the overall mechanical performance of the material [17,18]. In addition, gradient foams can serve as multilayer thermal insulators or sound-absorbing materials [19]. When the foam structure closely mimics that of bone tissue, it not only enhances mechanical strength but also facilitates cell attachment and proliferation, making it advantageous for biomedical applications [20,21].

In this study, a gradient in the cell density was generated by controlling the one-sided heating time, and a predictive model was developed by incorporating a physically meaningful cell coalescence function into classical nucleation theory (CNT). This approach allows researchers to tailor cell densities according to specific application requirements, demonstrating the potential to realize the various advantages described earlier.

## 2. Materials and Methods

### 2.1. Materials

Amorphous thermoplastic PMMA (EUNSUNG POLYTEC, Chungcheongbuk-do, Republic of Korea) was used in the form of sheets with a thickness of 1.1 mm. The PMMA sheets were prepared as rectangular samples with dimensions of 10 mm × 40 mm to ensure proper placement inside the batch chamber. The density of the specimens was 1.19 g/cm^3^, and the glass transition temperature (T_g_) was 120 °C [22]. High-purity CO_2_ (99.9%; 40 L; Samheung GasTech, Seoul, Republic of Korea) was used as the blowing agent to achieve efficient and stable saturation within a short period during the MCP. Although N_2_ is commonly used as a blowing agent in injection molding, CO_2_ was selected in this study to achieve higher solubility. CO_2_ allows a greater amount of gas to dissolve into the polymer matrix than N_2_, making it more suitable for the saturation process.

### 2.2. Microcellular Foaming Process

**Figure 1:**
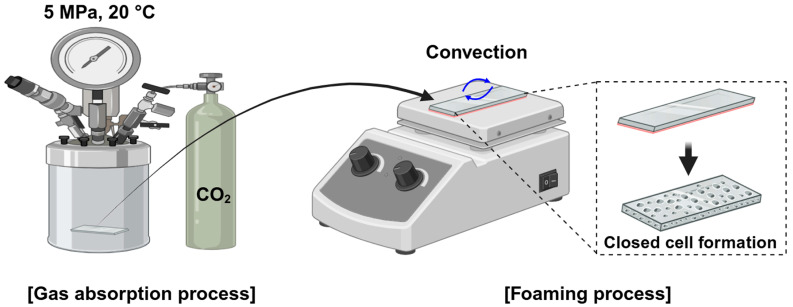
Schematic of the overall process of gradient cell density foaming. (Created in Biorender. Donghwan Lim. (2025) https://app.biorender.com).

To ensure stable implementation of the MCP, a solid-state batch foaming process was used. A batch chamber with an inner diameter of 50 mm and a depth of 50 mm was placed in an environment maintained at an atmospheric temperature of 20 °C. The PMMA sheet was placed inside the chamber, and CO_2_ was introduced at a pressure of 5 MPa for 4 h to allow sufficient gas dissolution into the PMMA sheet. After the saturation process, the pressure inside the chamber was reduced to atmospheric pressure via depressurization, and the PMMA sheet was then removed. Before removal, a hot plate (HSD-120, MISUNG SCIENTIFIC, Seoul, Republic of Korea) was preheated to 60 °C. The gas-absorbed PMMA sheet, which was removed from the batch chamber, was placed on the preheated hot plate to initiate foaming under one-sided heating conditions. The PMMA sample was clamped firmly onto the heating plate using a horseshoe-shaped metal jig to ensure full contact with the heating surface. The foaming temperature was set to 60 °C because the dissolution of CO_2_ into PMMA lowers its T_g_. After the 4 h absorption process, the T_g_ decreased below 40 °C, making 60 °C an appropriate temperature to induce controlled foaming without excessive cell growth, thereby allowing for clear observation of cell behavior [23,24]. The overall MCP procedure involving one-sided heating is illustrated in Figure 1, and the specific MCP parameters are summarized in Table 1.

### 2.3. Modeling

#### 2.3.1. Gas Absorption Diffusion Theory

**Figure 2:**
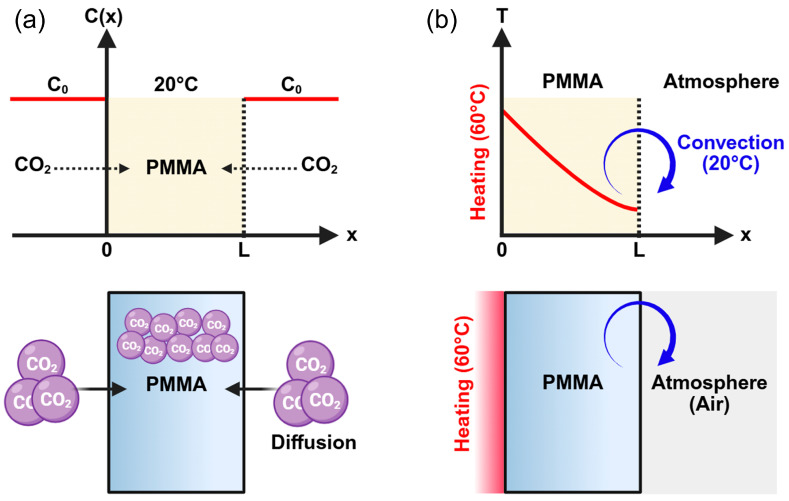
Schematic of diffusion (**a**) and temperature profiles (**b**). (Created in Biorender. Donghwan Lim. (2025) https://app.biorender.com).

The dissolution of CO_2_ into PMMA can be described as a diffusion process, and various diffusion models under different conditions have been established by J. Crank [25]. Diffusion can be modeled using Fick’s second law (Equation (1)). Assuming that gas diffusion occurs from both surfaces of the PMMA sheet and that diffusion in the thickness direction is negligible (Figure 2a), gas weight gain can be analytically calculated under these conditions. Based on the above assumptions, the boundary conditions are defined as $C\left(L,t\right)=C_{0}$ and $C\left(0,t\right)=C_{0}\;$, and the initial condition is given by $C\left(x,0\right)=0$.

(1) $$\frac{\partial C}{\partial t}=D\frac{\partial^{2}C}{\partial x^{2}}$$

By applying the boundary and initial conditions to Equation (1), the partial differential equation can be solved analytically. Consequently, the gas concentration profile can be expressed as follows:

(2) $$C\left(x,t\right)=C_{0}-\sum_{n=1}^{\infty }\frac{4C_{0}}{\left(2n-1\right)\pi }\cdot \sin\left(\frac{\left(2n-1\right)\pi }{L}x\right)exp(-D{\left(\frac{\left(2n-1\right)\pi }{L}\right)}^{2}t)$$

where *C(x,t)* denotes the CO_2_ concentration within the PMMA sheet as a function of the distance x through the thickness and time t; *C*_0_ denotes the CO_2_ concentration outside the PMMA sheet, *L* represents the thickness of the PMMA sheet, *t* represents the gas absorption time, and *D* represents the diffusion coefficient. By integrating Equation (2) over the entire thickness, the gas weight gain at an arbitrary time *t*, denoted as *M(t)*, can be calculated, as shown in Equation (3), where *M(∞)* represents the maximum weight gain, corresponding to solubility.

(3) $$\frac{M(t)}{M(\infty )}=1-\frac{8}{\pi^{2}}\sum_{n=1}^{\infty }\frac{1}{{\left(2n-1\right)}^{2}}\cdot exp(-D{\left(\frac{\left(2n-1\right)\pi }{L}\right)}^{2}t),\;n=1,2,3,\ldots$$

Computing an infinite number of terms for *n* is impractical; therefore, the series was limited to *n* = 20. The associated error when using 20 terms is less than 1%, which justifies the simplification for computational efficiency [26]. This approach allows for rapid and accurate estimation of gas weight gain.

#### 2.3.2. Sanchez–Lacombe Equation of State

The Sanchez–Lacombe equation of state (SL-EOS) (Equation (4)) was used to calculate the density of the PMMA and CO_2_ mixture within the batch chamber. As the CO_2_ dissolves into the PMMA sheet to form a mixture, direct measurement of the mixture density under high-pressure conditions inside the batch chamber is not feasible; therefore, the SL-EOS was applied for this purpose [27,28]. The equation involves the characteristic pressure ($P^{*}$), characteristic density ($\rho^{*}$), characteristic temperature ($T^{*}$), reduced density ($\tilde{\rho }$), reduced pressure ($\tilde{P}$), and reduced temperature ($\tilde{T}$), which are related as follows:

(4) $$\tilde{\rho }=1-exp[-\frac{{\tilde{\rho }}^{2}}{\tilde{T}}-\frac{\tilde{P}}{\tilde{T}}-\left(1-\frac{\phi_{1}}{r_{1}}\right)\tilde{\rho }],$$

(5) $${\tilde{P}}_{i}=\frac{P}{{P_{i}}^{*}},\;{\tilde{\rho }}_{i}=\frac{\rho }{{\rho_{i}}^{*}},\;{\tilde{T}}_{i}=\frac{T}{{T_{i}}^{*}},$$

(6) $$P^{*}=\phi_{1}{P_{1}}^{*}+\phi_{2}{P_{2}}^{*}-\phi_{1}\phi_{2}{∆P}^{*},$$

(7) $${∆P}^{*}={P_{1}}^{*}+{P_{2}}^{*}-{2\psi \left({P_{1}}^{*}{P_{2}}^{*}\right)}^{0.5},$$

(8) $$T^{*}=\frac{P^{*}}{\frac{\phi_{1}{P_{1}}^{*}}{{T_{1}}^{*}}+\frac{\phi_{2}{P_{2}}^{*}}{{T_{2}}^{*}}}$$

where subscript 1 represents CO_2_, subscript 2 represents PMMA, and the absence of a subscript denotes the PMMA–CO_2_ mixture; $\phi_{1}$ denotes the volume fraction of CO_2_ dissolved in the mixture, and $r_{1}$ denotes the number of lattice sites occupied by a CO_2_ molecule; ψ denotes the binary interaction parameter between CO_2_ and PMMA, and its value has been experimentally determined to be 0.975 [29]. Using Equations (4)–(8), the density of the PMMA–CO_2_ mixture can be calculated. This density is critical for calculating the surface interfacial tension, which is a key parameter in nucleation theory. When the gas weight gain is unknown, it is necessary to include the chemical potential equation for accurate calculation. However, in this study, a fully saturated PMMA–CO_2_ mixture was used, allowing the volume fraction of CO_2_ ($\phi_{1}$) to be fixed at a constant value of 0.2. This assumption simplifies SL-EOS modeling. The characteristic parameters for CO_2_ and PMMA used in the SL-EOS calculations are listed in Table 2.

#### 2.3.3. Temperature Profile Modeling

To analyze the foaming behavior induced by the one-sided heating of the gas-absorbed PMMA sheet, it is essential to evaluate the evolution of the internal temperature of the PMMA sheet. As illustrated in Figure 2b, the heated side is subjected to conduction at 60 °C, whereas the opposite side is exposed to convection under ambient conditions at 20 °C. Under these boundary conditions, the internal temperature profile of the PMMA sheet can be calculated as a function of time using the finite difference method (FDM). The unsteady heat conduction can be defined by the following partial differential equation:

(9) $$\frac{\partial T}{\partial t}=\alpha \frac{\partial^{2}T}{\partial x^{2}}.$$

where α denotes thermal diffusivity. The boundary conditions are defined as *T* = 60 °C *at x* = 0 (conduction) and $-\kappa \frac{\partial T}{\partial x}=h\left(T_{N}-T_{\infty }\right)\mathrm{at}x=L$ (convection). Given the complexity introduced by the convective boundary condition, an analytical solution was not feasible. Instead, the problem was approached numerically using the FDM. The final formulation is expressed in Equation (10), and the temperature distribution can be computed numerically using Python 3.12.10.

(10) $$T_{N-1}^{n}=T_{N}^{n}\left(1+\frac{h∆x}{\kappa }\right)-\frac{h∆x}{\kappa }T_{\infty }.$$

where *h* (W/m^2^ · K) denotes the convective heat transfer coefficient, and *κ* (W/m · K) denotes thermal conductivity.

#### 2.3.4. Classical Nucleation Theory

CNT is expressed in the form of Equation (11) without the inclusion of parameters A and B. This formulation was originally defined by early researchers in the field of microcellular foaming and has been extensively adopted [3]. This formulation assumes that microcells are generated homogeneously and has been validated in numerous studies. Equation (11) represents the nucleation rate, which indicates the number of nucleation events occurring per second. To calculate the actual cell density, the nucleation rate was integrated over the vitrification time (*t_vit_*), as shown in Equation (14). The surface interfacial tension ($\gamma_{mix}$) reflects the interaction between PMMA and CO_2_ and plays an essential role in describing both cell growth and collapse, making it a key factor in CNT. Equation (12) was used to determine this value [31]. However, the surface interfacial tension within the batch chamber could not be measured due to the high-pressure environment. Therefore, to compensate for the inaccuracy of Equation (12), a correction factor (B) was introduced to adjust the homogeneous nucleation activation energy, resulting in the modified expression shown in Equation (13).

(11) $$N_{hom}=Af_{0}C_{0}exp(\frac{{-{\Delta G}^{*}}_{hom}}{kT}B),$$

(12) $$\gamma_{mix}=\gamma_{PMMA}{\left(\frac{\rho_{mix}}{\rho_{PMMA}}\right)}^{4}{\left(1-\omega_{CO_{2}}\right)}^{4},$$

(13) $${{\Delta G}^{*}}_{hom}=\frac{16\pi {\gamma_{mix}}^{3}}{3{\Delta P}^{2}},$$

(14) $$N_{total}=\int_{0}^{t_{vit}}N_{hom}dt$$

where $f_{0}$ denotes the frequency at which CO_2_ molecules dissolved in PMMA contribute to nucleation per second. In the case of homogeneous cell formation, a typical value of approximately 10^−5^ $\left(s^{-1}\right)$ is used. However, due to the difficulty in determining the exact value, it is typically replaced or estimated using experimental data [31,32,33,34]. The value of $\gamma_{PMMA}$ was set to 0.0377 J/m^2^ based on the experimental data reported in previous studies [35].

At the early stage of foaming, nucleated CO_2_ receives thermal energy and begins to grow, resulting in the coalescence of adjacent cells. This physical phenomenon can be described using a function denoted as A. When a large amount of energy was absorbed and the concentration of dissolved CO_2_ in PMMA was high, the viscosity and T_g_ decreased, promoting cell growth and coalescence [23,36]. In contrast, in regions farther from the heat source, where less energy was absorbed and CO_2_ gradually desorbed from the PMMA over time, the viscosity increased. In such regions, cell growth becomes dominant over coalescence, resulting in a lower cell density. This spatial variation enabled the formation of a gradient cell density structure.

## 3. Results and Discussion

### 3.1. CO_2_ Weight Gain

A 1.1 mm-thick PMMA sample was saturated with CO_2_ at a pressure of 5 MPa in the batch chamber, and the weight gain was measured at various gas absorption times (30, 60, 120, and 180 min). An Ohaus electronic balance (Model No. AR2130) with a precision of 0.01 g was used to measure the weight of the polymer before ($W_{before}$) and after ($W_{after}$) saturation. The weight gain (%) was calculated using Equation (15).

(15) $$Weight\;gain\;(\%)=\frac{W_{after}\;\left(g\right)-W_{before}\;\left(g\right)}{W_{before}\;\left(g\right)}\times 100.$$

**Figure 3:**
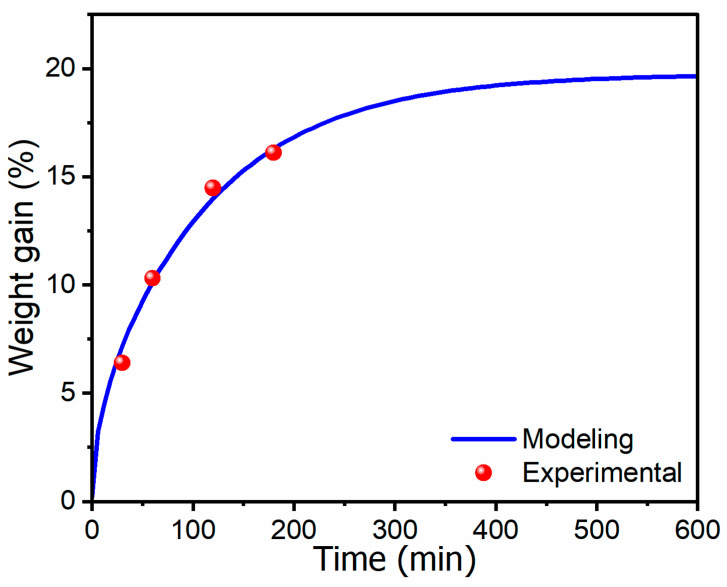
Curve fitting graph modeling gas absorption by fitting experimental data, showing the relationship between gas absorption time and weight gain, along with actual experimental values.

By applying the weight gain values obtained from Equations (15) to (3), numerical fitting was performed using Python 3.12.10 to determine the diffusion coefficient and solubility at 20 °C and 5 MPa (Figure 3). The numerically calculated value of M(∞) was 19.74%, and the diffusion coefficient was calculated to be 1.04 × 10^−9^ m^2^/min.

### 3.2. Temperature Profile

**Figure 4:**
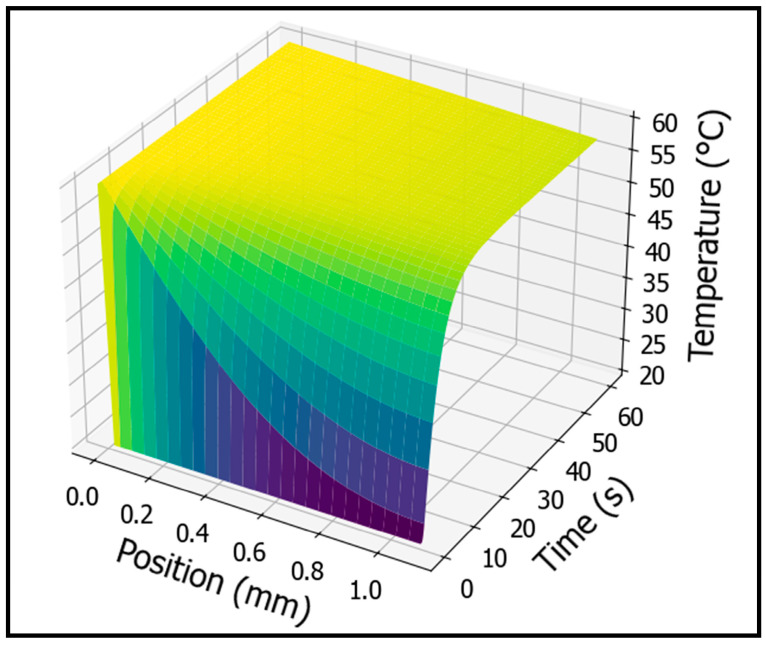
3D graph illustrating variations in the internal temperature profile of PMMA as a function of one-sided heating time. Blue indicates a temperature of 20 °C, while yellow represents 60 °C.

The internal temperature profile of PMMA during one-sided heating at 60 °C was numerically calculated using Equation (10) in Python 3.12.10. This allows the prediction of the evolution of the temperature profile over time during foaming and enables the incorporation of temperature into cell nucleation theory to obtain an accurate nucleation rate. As shown in Figure 4, a steep temperature gradient is observed at the initial stage of heating, which gradually transitions toward a steady-state profile resembling a linear function as time passes. In other words, using the graph in Figure 4, the internal temperature profile of PMMA at any given heating time can be identified, and the corresponding cell nucleation rate can be calculated to predict the actual foaming behavior.

### 3.3. Gradient Cell Density Analysis

#### 3.3.1. Cell Density Measurements

To measure the cell density, the foamed PMMA samples were immersed in liquid nitrogen (−196 °C) and then fractured to expose their cross-sections. The surfaces of the fractured samples were coated using an ion sputter coater (E1030, Hitachi, Tokyo, Japan) for 60 s. The prepared cross-sections were then imaged using a field-emission scanning electron microscope (FE-SEM, Nova Nano SEM 200, FEI, OR, USA). The acquired images were analyzed using ImageJ software 1.54p to determine the cell diameter and density. Cell density was calculated using Equations (16) and (17).

**Figure 5:**
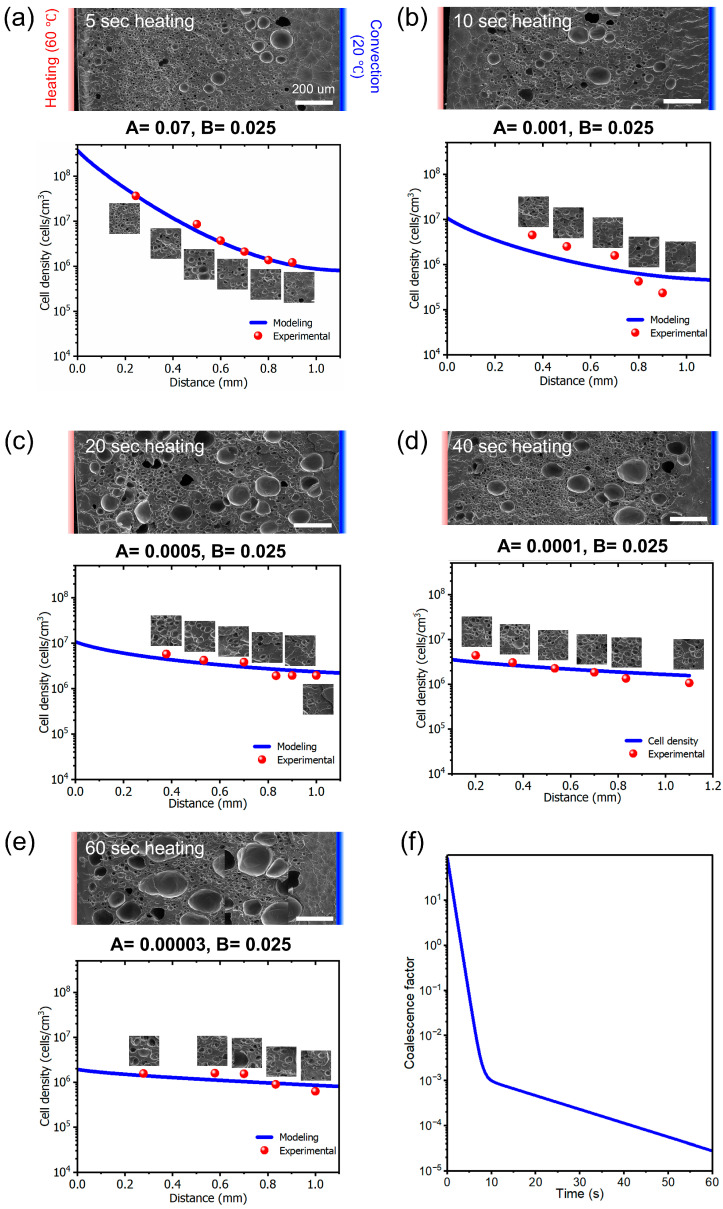
Experimental cell densities extracted from FE-SEM images of PMMA cross-sections at different one-sided heating times, along with the corresponding modeling curves (code S1). Parameters A and B represent the correction function and factor, respectively, used in the cell nucleation rate equations for each heating duration. The red bar indicates the heated region, while the blue bar represents the surface exposed to air convection. (**a**) 5, (**b**) 10, (**c**) 20, (**d**) 40, and (**e**) 60 s heating. (**f**) Graph of coalescence factor representing the extent of cell coalescence as a function of heating time.

However, accurate cell density measurements require consideration of the expansion ratio, which is difficult to determine because of the gradient foam structure. Therefore, porosity was calculated directly from the FE-SEM images, and it was assumed that the 2D porosity approximates the 3D porosity [37]. This assumption enabled the estimation of cell density from the FE-SEM data.

(16) $$\mathrm{Average}\mathrm{cell}\mathrm{diameter}\left(\mu m\right)=\frac{\sum d_{i}n_{i}}{\sum n_{i}},$$

(17) $$Celldensity({cm}^{-3})={\left(\frac{n_{b}M^{2}}{A}\right)}^{\frac{3}{2}}\times \frac{1}{\epsilon }.$$

where d_i_ denotes the diameter of cell type i, n_i_ denotes the number of cells of type i, n_b_ denotes the total number of bubbles in the micrograph, M represents the magnification factor, A represents the surface area of the micrograph, and ε represents porosity. The FE-SEM images used for experimental analysis at different foaming times are shown in Figure 5.

#### 3.3.2. Effect of Cell Coalescence

As previously mentioned, following cell nucleation, a rapid temperature increase leads to a decrease in viscosity, thinning of the cell walls, and eventual collapse, resulting in cell coalescence. This phenomenon occurs within a short time after heating. Subsequently, gas desorption increases viscosity, thereby reducing the rate of cell coalescence, promoting cell growth, and increasing cell size. Therefore, by analyzing the FE-SEM images at each heating time and determining the corresponding modeled values of A and B, a unified cell coalescence function (*A*) was established to model the entire system. The time-dependent cell coalescence function is expressed in Equation (18).

(18) $$A\left(t\right)=84.5419\;e^{-1.4232t}+0.0019079\;e^{-0.07034t}$$

In physical terms, this indicates that cell coalescence occurs rapidly during the initial stage of heating and gradually slows down as the heating time increases (Figure 5f).

## 4. Conclusions

In this study, a cell density gradient was experimentally achieved by fully saturating PMMA with CO_2_ gas through a solid-state batch foaming process, followed by one-sided heating. The experimentally obtained gradient was then used to establish and validate a theoretical framework for cell coalescence based on a modified classical nucleation theory (CNT). When extended to incorporate the physical properties of various polymers, the developed model has the potential to predict cell density gradients through simple and accessible experimental procedures beyond PMMA systems.

Unlike previous approaches, this method offers a simplified yet effective way to generate gradient cellular structures. By fully saturating the polymer with gas and applying heat to one side, a thermal gradient is introduced via conduction, which in turn induces a cell density gradient. Unlike earlier methods that required delicate control over multiple parameters to generate such gradients, the current approach requires only precise control of heating time and temperature to reproduce the cell distribution predicted by the theoretical model. In addition, by tailoring mechanical properties such as impact strength and elastic modulus through spatial cell variation, materials can be customized to meet specific functional requirements. In biomedical applications, this technique holds great promise for fabricating bone scaffolds with gradient porosity that mimics the natural density distribution of bone tissue, offering potential advantages in the treatment of bone-related diseases.

However, this study did not incorporate a temperature prediction model that accounts for the internal thermal profile of CO_2_-saturated PMMA during one-sided heating. This omission was due to the difficulty of predicting the thermal behavior of the polymer when gas molecules are absorbed into the free volume between polymer chains, which significantly alters the mechanical and thermal properties compared to those of the neat polymer. Furthermore, currently available simulation tools are limited in their ability to capture such gas–polymer interactions. Therefore, the temperature distribution was modeled without considering gas absorption. Despite this simplification, the experimentally observed cell behavior exhibited good agreement with the predictions, validating the use of the conventional heat conduction model.

Another limitation was the thinness of the PMMA sheet, which made it difficult to finely control the internal temperature profile. Although the use of thicker PMMA sheets could enable more accurate modeling and prediction of the temperature gradient, it would also significantly increase the saturation time required for gas absorption, making the experimental process more time-consuming. As part of future work, thicker PMMA sheets will be employed to more precisely predict and measure the internal temperature profile and confirm the formation of a gradient cell structure. Furthermore, mechanical characterization of PMMA specimens with gradient cell densities will be performed and compared with that of PMMA with homogeneous cell structures, in order to explore the effects of spatial cell variation on mechanical performance.

## Figures and Tables

**Table 1:** Experimental parameters of the MCP.

Property	Value
Saturation pressure (MPa)	5 ± 0.2
Saturation temperature (°C)	20 ± 2
Saturation time (h)	4
Average depressurization rate (MPa/s)	2
Heating temperature (°C)	60
Heating time (s)	5, 10, 20, 40, 60

**Table 2:** Characteristic parameters of PMMA and CO_2_ in SL-EOS [30].

Subscript	Component	$P^{*}$	$T^{*}$	$\rho^{*}$
1	CO_2_	574.5	305.3	1.510
2	PMMA	500.1	741.3	1.247

## Data Availability

The original contributions presented in this study are included in the article. Further inquiries can be directed to the corresponding author.

## Supplementary Materials

The following supporting information can be downloaded at: https://www.mdpi.com/article/10.3390/polym17131780/s1, Code S1: cell density modeling code.

## Abbreviations

The following abbreviations are used in this manuscript:

FE-SEM	Field-emission scanning electron microscope
FDM	Finite difference method
SL-EOS	Sanchez–Lacombe equation of state
