# Supplementary material for: Modeling and Experimental Validation of Gradient Cell Density in PMMA Microcellular Foaming Induced by One-Sided Heating
Source: Polymers (Basel). 2025 Jun 27;17(13):1780. doi: 10.3390/polym17131780 (PMC12252372; doi:10.3390/polym17131780)
Supplement: Supplementary file 1 [file polymers-17-01780-s001.zip › polymers-3713230-supplementary.pdf]

Supplementary

S1. Cell density calculation python code

```
import numpy as np
import matplotlib.pyplot as plt
from scipy.optimize import fsolve
import math

k_heat   = 0.15      # W/(m·K)
h_conv   = 10.0      # W/(m²·K)
L        = 0.0011    # m
alpha    = 1.09e-7   # m²/s
T_inf    = 20.0      # °C
T_left   = 60.0      # °C

Nx       = 50
dx       = L / Nx
dt_max  = 0.5 * dx**2 / alpha
dt       = 0.8 * dt_max
t_final = 5.0
steps   = int(t_final / dt)
time_arr = np.linspace(0, t_final, steps+1)

print("Selected dt =", dt, "s")

T_all = np.ones((steps+1, Nx+1)) * T_inf
T_all[0, 0] = T_left
T_profile = T_all[0].copy()
r_heat = alpha * dt / dx**2

for n in range(steps):
```

```
    T_new = T_profile.copy()
    for i in range(1, Nx):
        T_new[i] = T_profile[i] + r_heat * (T_profile[i+1] - 2*T_profile[i] + T_profile[i-1])
    # Dirichlet at x=0
    T_new[0] = T_left
    # Robin at x=L
    T_new[Nx] = (T_new[Nx-1] + (h_conv*dx/k_heat)*T_inf) / (1 + (h_conv*dx/k_heat))
    T_profile = T_new
    T_all[n+1] = T_profile

x_space = np.linspace(0, L, Nx+1) * 1000  # mm

R_gas = 8.314
P_star_PMMA = 500.16
T_star_PMMA = 741.38
rho_star_PMMA = 1.247
rho_PMMA = 1.17
P_star_CO2 = 720.3
T_star_CO2 = 269.5
rho_star_CO2 = 1.58
psi = 0.975
r_CO2 = P_star_CO2 * 44 / (R_gas * T_star_CO2 * rho_star_CO2)
N_av = 6.022e23
k_B = 1.380649e-23

def weight_to_volume_fraction(w_CO2, rho_star_polymer, rho_star_CO2):
    ratio = (w_CO2 / (1 - w_CO2)) * (rho_star_polymer / rho_star_CO2)
    return ratio / (1 + ratio)

def calculate_rho_reduced(phi_CO2, T_reduced, P_reduced, r_CO2):
    def eq(x): return 1 - np.exp(-x**2/T_reduced - P_reduced/T_reduced - (1 - phi_CO2/r
```

```
_CO2)*x) - x
    return fsolve(eq, 0.8)[0]


def compute_gamma_mix(P, w_CO2_fixed, T_local):
    phi_CO2 = weight_to_volume_fraction(w_CO2_fixed, rho_star_PMMA, rho_star_C
O2)
    phi_PMMA = 1 - phi_CO2
    P_star_mix = (phi_PMMA*P_star_PMMA + phi_CO2*P_star_CO2
                  - phi_PMMA*phi_CO2*(P_star_PMMA + P_star_CO2 - 2*psi*np.sqrt(P_st
ar_PMMA*P_star_CO2)))
    T_star_mix = P_star_mix / (phi_PMMA*P_star_PMMA/T_star_PMMA + phi_CO2*
P_star_CO2/T_star_CO2)
    T_red = T_local / T_star_mix
    P_red = P / P_star_mix
    rho_red = calculate_rho_reduced(phi_CO2, T_red, P_red, r_CO2)
    rho_star_mix = 1 / (w_CO2_fixed/rho_star_CO2 + (1-w_CO2_fixed)/rho_star_PMM
A)
    rho_mix = rho_red * rho_star_mix
    gamma = 0.039 * (rho_mix/rho_PMMA)**4 * ((1-w_CO2_fixed)**4)
    return gamma


def nucleation_rate(t, A_in, B_in, Tf_local, w_CO2_fixed=0.18):


    A = 0.07
    B = 0.025
    P = 5.0  # MPa
    rho_CO2_local = 0.1  # g/cm³
    gamma_mix = compute_gamma_mix(P, w_CO2_fixed, Tf_local)
    delta_G = (16*np.pi*gamma_mix**3) / (3*((P-0.1)*1e6)**2)
    C = rho_CO2_local * N_av / 44
    return 1e-5 * A * C * np.exp(-delta_G * B / (k_B * Tf_local))
```

```
cell_density = np.zeros((steps+1, Nx+1))

for i in range(Nx+1):
    cum = 0.0
    for n in range(steps+1):
        Tf = T_all[n, i] + 273.15
        rate = nucleation_rate(time_arr[n], None, None, Tf)
        if n > 0:
            cum += rate * (time_arr[n] - time_arr[n-1])
        cell_density[n, i] = cum

plt.figure(figsize=(8,6))
plt.plot(x_space, cell_density[-1], marker='o')
plt.xlabel("Position (mm)")
plt.ylabel("Cell Density")
plt.title(f"Cell Density vs. Thickness at t = {t_final:.1f} s (A=B=1)")
plt.yscale('log')
plt.grid(True)
plt.show()
```
